# Preoperative contrast-enhanced ultrasound combined with intra-lymph node methylene blue injection for sentinel lymph node identification: a minimally invasive sentinel lymph node biopsy tracing approach

**DOI:** 10.1007/s00330-025-11932-3

**Published:** 2025-08-21

**Authors:** Yang Liu, Jian Wu, Wenjie Zhang, Tielin Wang, Shuang Wu, Hong Zhou, Yang Zhou, Ying Liu

**Affiliations:** 1 Department of Ultrasound, Affiliated Hospital of Southwest Jiaotong University, The Third People’s Hospital of Chengdu, Chengdu, China; 2 Department of Breast and Thyroid Surgery, Affiliated Hospital of Southwest Jiaotong University, The Third People’s Hospital of Chengdu, Chengdu, China

**Keywords:** Breast cancer, Contrast agent, Ultrasonography, Sentinel lymph node biopsy

## Abstract

**Objectives:**

To investigate the significance of preoperative contrast-enhanced ultrasound (CEUS) coupled with injections of methylene blue (MB) into the lymph nodes, alongside intracutaneous injections of indocyanine green (ICG) for sentinel lymph node (SLN) identification in early breast cancer.

**Materials and methods:**

All patients from a single institution were prospectively randomized into two groups: CEUS Group (preoperative SLN-CEUS coupled with injections of MB into the lymph nodes, with ICG intracutaneous injections for SLN identification during surgery) and Blue Staining Group (intracutaneous injections of both ICG and MB for SLN mapping during surgery). Pathological results served as the gold standard. Multivariate logistic regression analysis was used to identify independent risk factors for SLN metastasis. Areas under the receiver-operating characteristic curve (AUC) were used to evaluate the ability of CEUS to diagnose SLN metastasis.

**Results:**

134 patients were enrolled (CEUS Group: 76 patients, Blue Staining Group: 58 patients). CEUS Group achieved a 100.0% success rate in detecting SLNs, with identifying a median of 1 SLN, while Blue Staining Group identifying a median of 4 SLNs per case (*p* < 0.001). CEUS enhancement pattern was the only factor independently linked to SLN metastasis (*p* < 0.001), showing a sensitivity of 90.5% and a specificity of 94.5%. The AUC for identifying SLN metastasis was 0.925.

**Conclusions:**

CEUS enhancement pattern is helpful to determine the SLN metastasis. Preoperative CEUS, combined with MB injections into lymph nodes and intracutaneous ICG injections during surgery, provides a reliable method for localizing SLNs. This approach minimizes the risk of non-SLNs excisions.

**Key Points:**

***Question*** *Despite the critical role of sentinel lymph node (SLN) detection in surgical outcomes, current preoperative imaging-guided SLN tracing remains in the exploratory stage.*

***Findings*** *Contrast-enhanced (CE) US achieved 100% SLN detection success with fewer non-target lymph nodes excised versus blue dye, demonstrating superior sensitivity and specificity metastatic prediction.*

***Clinical relevance*** *Preoperative SLN-CEUS, combined with intralymphatic methylene blue injection and intracutaneous indocyanine green administration during surgery, enables precise SLN identification and metastasis prediction. This approach minimizes the risk of unnecessary non-SLN resection during SLNB.*

**Graphical Abstract:**

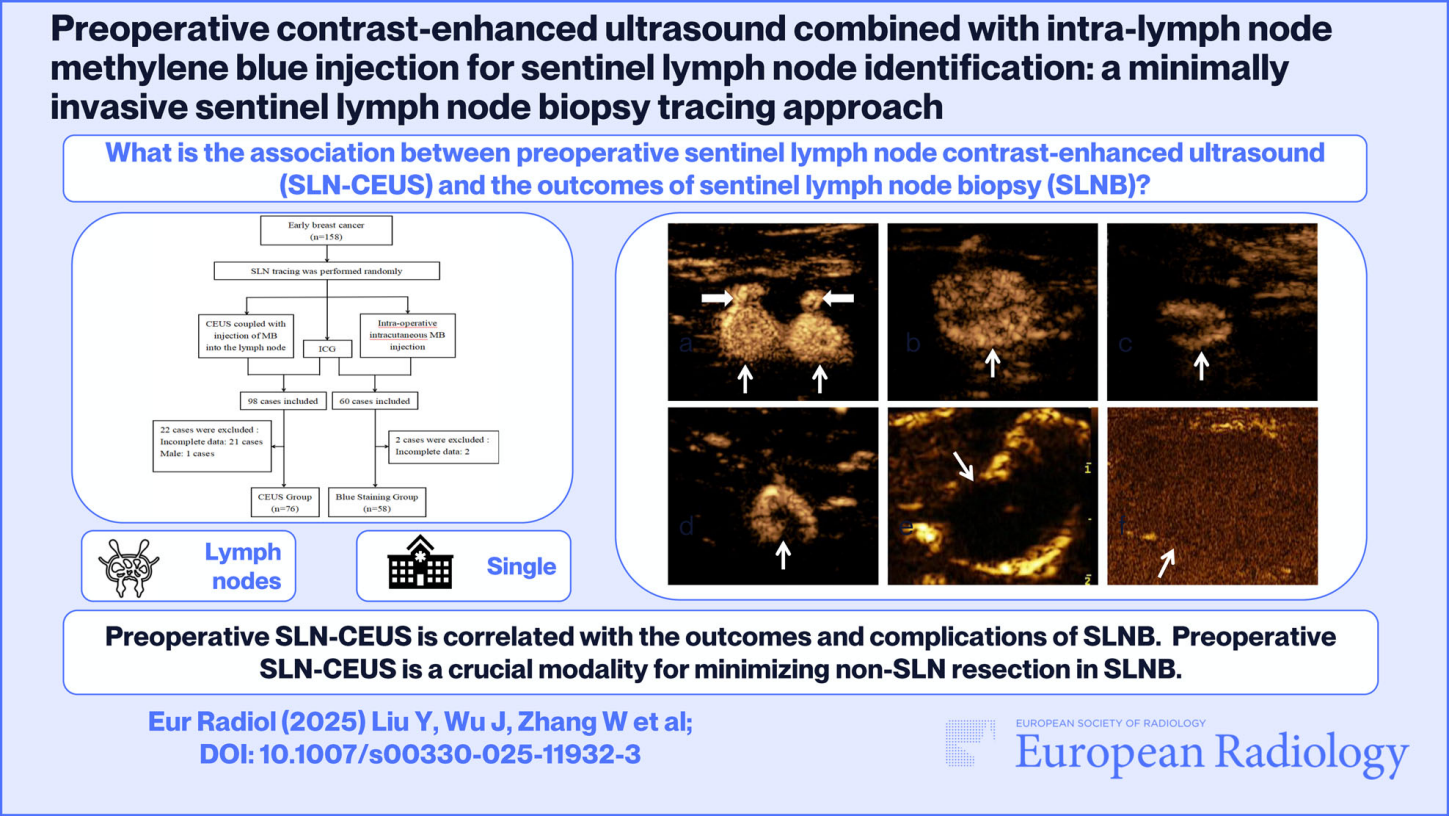

## Introduction

Breast cancer has surpassed lung cancer to emerge as the most prevalent malignancy affecting women’s health, significantly impacting their quality of life [1–4]. Axillary lymph nodes (ALNs) serve as the primary drainage route for breast cancer, and their status is a crucial prognostic indicator that informs overall survival and guides clinical adjuvant treatment decisions. Although axillary lymph node dissection (ALND) remains the standard of care for patients with positive lymph nodes, it is associated with several complications, such as upper limb lymphedema, arm paresthesia, and restricted shoulder mobility [5]. The sentinel lymph node (SLN) is the first lymph node or group of lymph nodes to receive lymphatic drainage from the primary tumor via the corresponding lymphatic channel (LC). It acts as a reliable indicator of the ALNs status in breast cancer, given that skip metastases are uncommon in this disease [6]. The National Comprehensive Cancer Network guidelines advocate for the use of sentinel lymph node biopsy (SLNB) as a replacement for ALND in clinically node-negative patients [7, 8]. This approach minimizes complications associated with ALND and aligns with the principles of precision medicine.

Accurate detection and localization of SLNs are vital for successful SLNB. In clinical practice, various tracer methods are employed to identify and locate LCs and SLNs, with blue dye (BD) and radioisotope (RI) tracing being the two most prevalent approaches [9]. Methylene blue (MB), due to its affordability and wide availability, is the most commonly used BD for SLNB. While MB is user-friendly, this dye has several limitations: it may cause skin discoloration, trigger allergic reactions in certain individuals, and has a tendency to yield a high false negative rate (FNR) [10]. The RI tracer method is effective, but its high cost, risks from radioactivity, and the need for specialized medical equipment limit its use, especially in primary healthcare settings. Recently, indocyanine green (ICG)-fluorescent imaging has emerged as a viable alternative for SLN tracking. Multiple studies have demonstrated that ICG is equivalent to RI and superior to BD regarding the SLN identification rates [11, 12]. The advantage of ICG-fluorescent imaging lies in its simplicity and non-radioactive nature. However, it still has some limitations, including limited penetration depth and the risk of intra-operative extravasation that could contaminate the surgical field [9].

Both the BD tracer method and ICG-fluorescent imaging are limited to detecting SLNs during surgery, revealing a significant gap in preoperative evaluation. This limitation highlights the urgent need for a reliable and streamlined approach to assess SLNs before surgery, making SLNB as minimally invasive as possible with the aid of image guidance [13]. In recent years, contrast-enhanced ultrasound (CEUS) has emerged as a promising solution for preoperative SLN localization and assessment in breast cancer [14, 15]. By injecting an ultrasound contrast agent (UCA) intracutaneously, CEUS provides clear, real-time preoperative images that accurately identify SLNs and LCs, overcoming the limitations of existing methods [4, 16]. It also assists in evaluating SLN conditions and guiding axillary surgery. However, CEUS results can be operator-dependent and are not recommended for patients with restricted upper limb abduction. Despite the advantages of CEUS and ICG, single-tracer methods have significant limitations, including variable success rates and an increased risk of missing lymph nodes in complex anatomical variations, which can lead to false negatives [14, 17]. False negatives are particularly concerning as they can result in undetected metastasis, delaying necessary treatment and negatively impacting patient outcomes. Factors such as lymphatic obstruction or surgical manipulation can further contribute to partial lymph node escape and increase the FNR [18]. To address these challenges and improve detection rates while minimizing the FNR, adopting dual tracing methods in SLNB for breast cancer is critical. Integrating CEUS for preoperative assessment with ICG, known for its convenience and non-radioactive properties, presents a compelling solution for improving SLN detection and localization.

This study aims to investigate the efficacy of combining preoperative SLN-CEUS with MB injections into lymph nodes, along with intra-operative intracutaneous ICG injections. Together, these methods represent an innovative imaging modality for SLN detection.

## Materials and methods

### Patient selection and classification

**Fig. 1:**
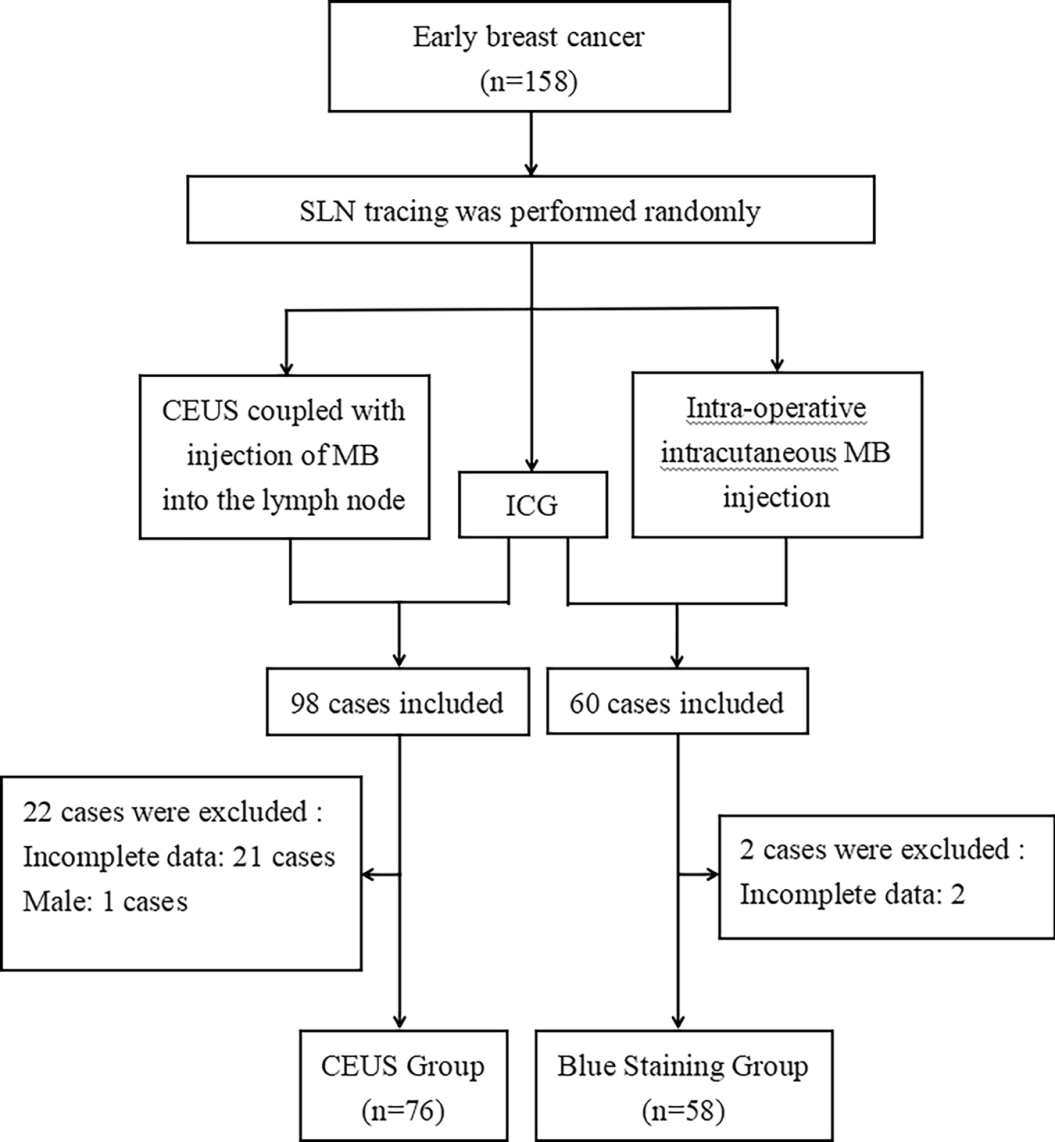
Flowchart of the study. SLN, sentinel lymph node; CEUS, contrast-enhanced ultrasound; ICG, indocyanine green; MB, methylene blue

This prospective study was approved by the Institutional Review Committee and Ethics Committee of the Third People’s Hospital of Chengdu, and the approval number Ethics Review (Research) No. [2024] S-44. All participants provided written informed consent prior to enrollment. From January 2022 to March 2024, a total of 158 patients with early breast cancer were enrolled at Chengdu Third People’s Hospital. All patients were randomly assigned to one of two groups: (1) CEUS Group, where preoperative CEUS was coupled with injections of MB into the lymph nodes, in conjunction with intracutaneous injection of ICG for SLN identification during surgery. (2) Blue Staining Group, where intracutaneous injections of ICG and MB were used for SLN mapping during the surgical procedure. Among the 158 patients, 98 patients were randomly assigned to the CEUS Group, and the remaining 60 were randomly assigned to the Blue Staining Group. The inclusion criteria for this study were as follows: (1) female gender, (2) pathologically confirmed breast cancer, (3) preoperative clinical staging of Tis, T1N0M0, or T2N0M0 breast cancer, (4) absence of palpable ALNs, and (5) eligibility for SLNB. Patients were excluded from the study if they had a previous history of axillary surgery, were in a pregnant or lactating state, exhibited hypersensitivity to UCA, or were diagnosed with inflammatory breast cancer. Following these criteria, 22 patients in the CEUS Group and 2 patients in the Blue Staining Group were excluded, leaving 76 and 58 eligible patients, respectively, to be included in the study. The flowchart is shown in Fig. 1.

### Instruments and materials

Patients in the CEUS Group using a GE-LOGIQ E11 (GE Healthcare) ultrasound system with CEUS tech. Instrumental parameters were optimized for lesion observation, with a mechanical index value of 0.09 to reduce microbubble damage. SonoVue lyophilized powder (Bracco Imaging SpA) was employed as the UCA and was mixed with 5 mL of normal saline. After shaking for > 20 s, a sulfur hexafluoride microbubble suspension was formed. MB (Jumpcan Pharmaceuticals) was used as the BD agent, and 1 mg of MB was extracted with a syringe for use.

### CEUS

**Fig. 2:**
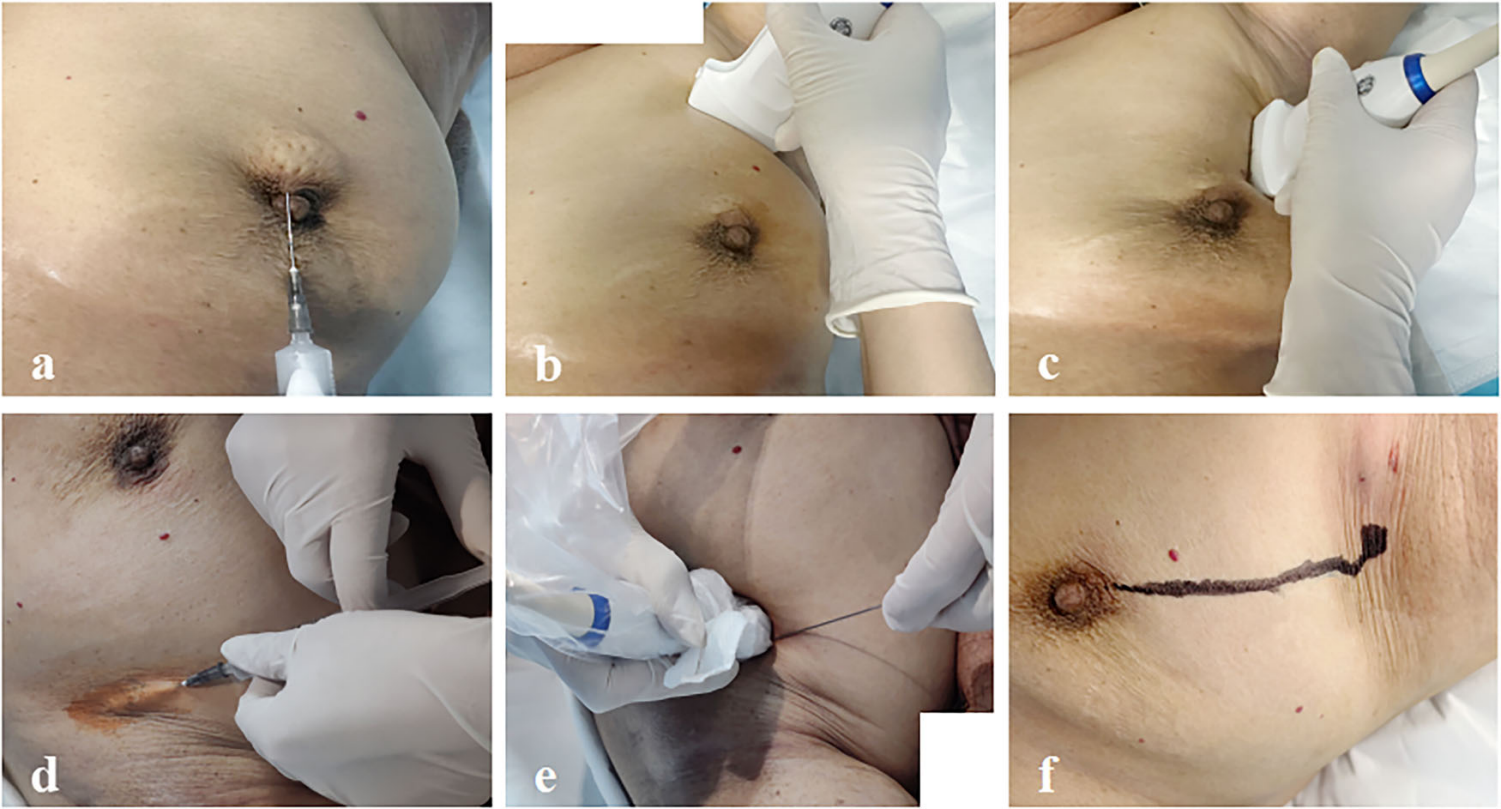
SLN-CEUS and intra-lymph node MB injection. Procedures: scanning, intra-lymph node MB injection, and marking. Following the disinfection of the areola and armpit: **a** Injection of contrast agent. **b** Acquisition of short-axis scans. **c** Acquisition of long-axis scans. **d** Injection of anesthetic. **e** Intra-lymph node MB injection. **f** Surface marking of LC and SLN. SLN, sentinel lymph node; CEUS, contrast-enhanced ultrasound; MB, methylene blue; LC, lymphatic channel

**Fig. 3:**
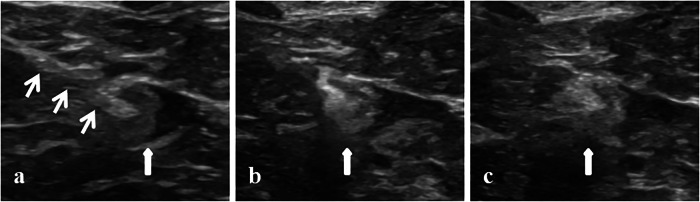
Sonographic monitoring of SLN changes following MB injection. The thin white arrows represent the puncture needle, the thick white arrow represents the SLN. **a** During puncture. **b** During injection. **c** After injection. SLN, sentinel lymph node; MB, methylene blue

The day prior to the SLNB, patients were placed in a supine position, with the affected breast and axillary region uncovered. After disinfection, at the 12 o’clock position on the areola, a 2.4 mL suspension of UCA was administered as a bolus through an intracutaneous injection. The 2–9 MHz linear array transducer in CEUS mode was carefully directed towards the axilla to trace the short axis of the enhanced LC. Once the LC became visible, the probe was rotated to reveal the long axis of the LC. This technique was repeated for each identified LC until the corresponding SLN was identified. The long and short diameters of the largest identified SLN and those of the CEUS-suspected SLN were measured and recorded. When CEUS interprets it as a non-suspicious SLN metastasis, the value is assigned as 0. Subsequently, the transducer was switched to conventional ultrasound (US) mode to guide the injection of MB into the identified SLNs. Finally, the pathways of the LCs and SLNs were marked on the patient’s body surface with a marker pen. All SLN-CEUS examinations were performed by a single physician with over six years of experience in SLN-CEUS procedures. Image analysis was conducted by two physicians, each with a decade of expertise in ultrasound imaging. The enhancement patterns of SLNs identified through CEUS were classified into three types: homogeneous, heterogeneous, and no enhancement, based on the extent and uniformity of the UCA distribution within the SLNs. Homogeneous enhancement is typically indicative of non-metastatic SLNs, while heterogeneous enhancement and the absence of enhancement are considered signs of metastatic SLNs [15, 19]. In instances where a patient exhibits multiple suspicious SLNs, the SLN with the greatest degree of suspicion was selected for analysis. The SLN-CEUS and US-guided intra-lymph node MB injection process, and sonographic monitoring of SLN changes following MB injection are shown in Figs. 2 and 3, respectively.

### Surgical procedure

The surgical process began with the preparation of an ICG solution (25 mg vial, Yichuang Pharmaceutical Co.) to a concentration of 1.0 mg/mL. After anesthetizing the patient in the same position as CEUS, 0.5 mL of MB (10 mg/mL) and 0.5 mL of ICG (1.0 mg/mL) were injected intracutaneously around the mass at four points. The area was then massaged for 10 min. Lymphatic flow was visualized using a fluorescence camera (SPY Portable Handheld Imaging System, NOVADAQ Stryker®). The SLNB incision site was determined by the disappearance of the fluorescent signal. MB-stained and fluorescently stained lymph nodes were excised for histological examination. Postoperative pathological results were the gold standard for comparing the number of SLNs detected between CEUS and Blue Staining Groups, and for evaluating the diagnostic accuracy within the CEUS Group.

### Pathological analysis

The harvested SLNs underwent routine histopathological examination at approximately 2-mm intervals. Immunohistochemistry was carried out to confirm any suspected metastases. All analyses were conducted by two pathologists, each with over a decade of diagnostic experience.

### Statistical analysis

SPSS 24.0 software package was utilized for statistical analysis. Continuous variable data were expressed as mean and standard deviation (± SD) or median and interquartile range (IQR); categorical variables were represented by the absolute value (*n*) and relative frequency (%). Continuous variables were compared between groups using the *t*-test or non-parametric tests. Categorical variables were analyzed with the Chi-square test. Physician diagnostic consistency was evaluated using Cohen’s kappa statistic. Spearman’s correlation was used for CEUS-pathology comparison. Multivariate logistic regression identified independent factors for SLN metastasis, expressed as ORs with 95% CIs. The performance of CEUS in diagnosing SLN status in early breast cancer was assessed using the receiver-operating characteristic curve (ROC) and area under the curve (AUC). Statistical significance was set at *p* < 0.05.

## Results

### Baseline characteristics

A total of 134 eligible female patients were enrolled in this study, including 76 patients in the CEUS Group and 58 patients in the Blue Staining Group. Table 1 illustrates that there were no statistically significant differences observed between the CEUS Group and the Blue Staining Group with respect to patient age (*p* = 0.81), lesion laterality (*p* = 0.71), lesion size (*p* = 0.79), and pathological type (*p* = 0.86). Each patient had a solitary breast lesion. Invasive ductal carcinoma was the predominant pathological type, comprising 72.4% (55/76) of cases in the CEUS Group and 69.0% (40/58) in the Blue Staining Group. Preoperative neoadjuvant chemotherapy (NAC) was administered to 18 patients in the CEUS Group and 11 patients in the Blue Staining Group, respectively.

**Table 1:** Characteristics of patients and tumors

Characteristics	CEUS group (*n* = 76)	Blue staining group (*n* = 58)	*p*-value
Age, years, mean ± SD	55.42 ± 10.84	54.98 ± 11.09	0.81
Laterality, *n* (%)			0.71
Left	43 (56.6)	31 (53.4)	
Right	33 (43.4)	27 (46.6)	
Tumor size, mm, mean ± SD	18.34 ± 11.50	17.84 ± 10.68	0.79
Pathology types, *n* (%)			0.86
Invasive ductal carcinoma	55 (72.4)	40 (69.0)	
Ductal carcinoma in situ	13 (17.1)	12 (20.7)	
Other	8 (10.5)	6 (10.3)	

*CEUS* contrast-enhanced ultrasound, *SD* standard deviation

### Number of SLNs

For the CEUS Group, the 76 patients undergoing SLNB had a total of 94 SLNs (averaging 1.66 ± 0.84 per patient, with a median of 1). In contrast, the Blue Staining Group’s 58 patients yielded 245 SLNs, averaging 4.22 ± 1.74 per patient, with a median of 4. Table 2 shows that the number of SLNs detected in the CEUS Group was significantly fewer than that in Blue Staining Group (z = 8.499, *p* < 0.001).

**Table 2:** Comparison of the number of SLNs detected between CEUS group and Blue staining group

Group	M (IQR)	Median difference (95% CI)	Wilcoxon two-sample rank sum test	
			z value	*p*-value
CEUS	1.00 (1)	2.00 (2.00–3.00)	8.499	< 0.001
Blue staining	4.00 (2)			

Data are shown as median (IQR)

*IQR* interquartile range, *CEUS* contrast-enhanced ultrasound, *SLN* sentinel lymph node, *CI* confidence interval, *z* Wilcoxon rank sum test

### Pathologic findings

In the CEUS Group, 32 of the 94 evaluated SLNs were metastatic in 21 patients, while 55 patients showed no signs of metastasis. In the Blue Staining Group, 23 of the 245 evaluated SLNs were metastatic in 15 patients, with 43 patients having no metastatic SLNs detected.

### CEUS findings

**Fig. 4:**
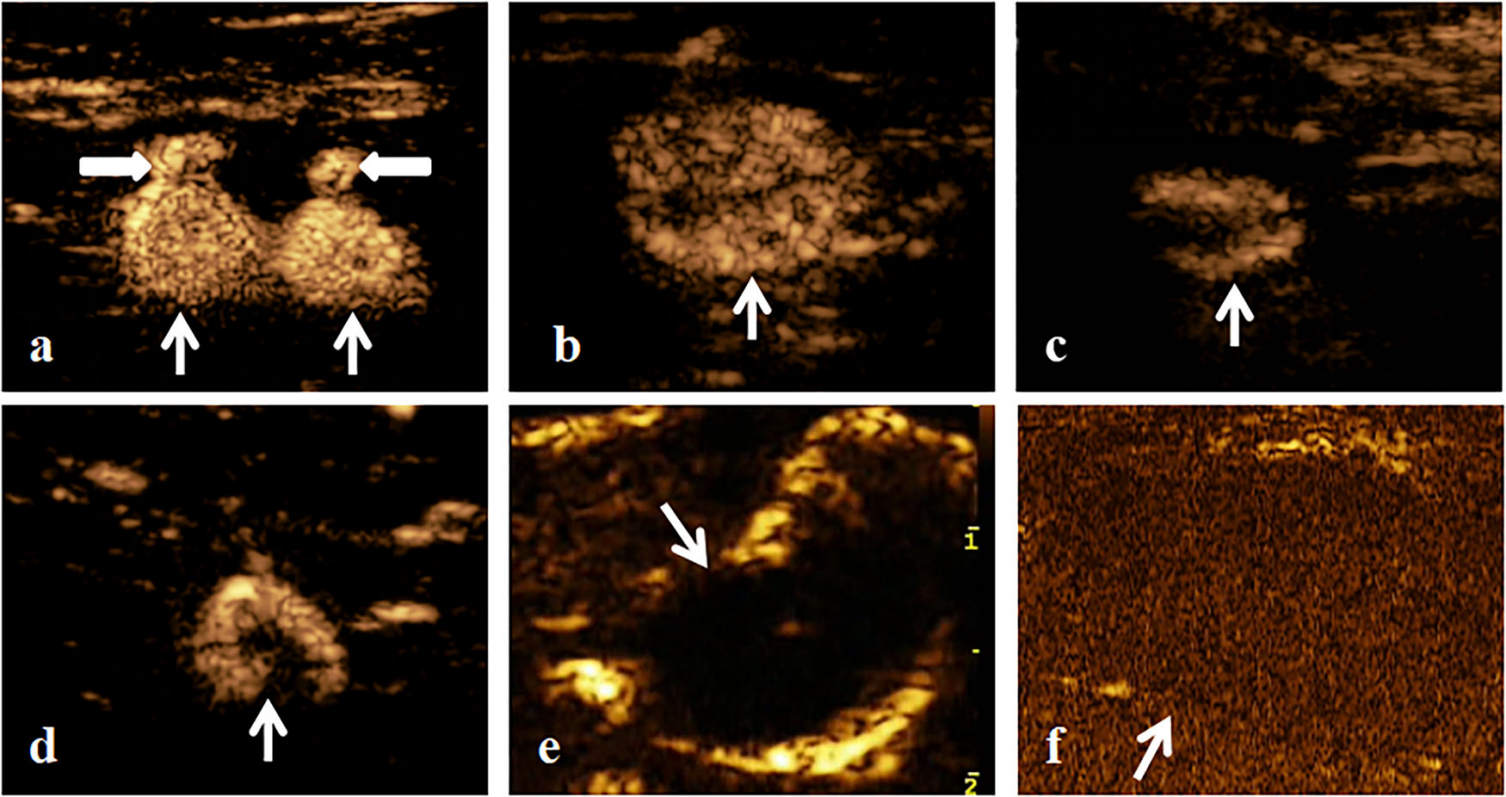
The CEUS enhancement patterns of SLN. The thin white arrows represent the SLN, the thick white arrows represent the short-axis section of LC: **a** Homogeneous enhancement. **b**–**e** Heterogeneous enhancement. **f** No enhancement. CEUS, contrast-enhanced ultrasound; SLN, sentinel lymph node; LC, lymphatic channel. Homogeneous enhancement indicates no SLN metastasis; heterogeneous or no enhancement suggests the presence of SLN metastasis

**Fig. 5:**
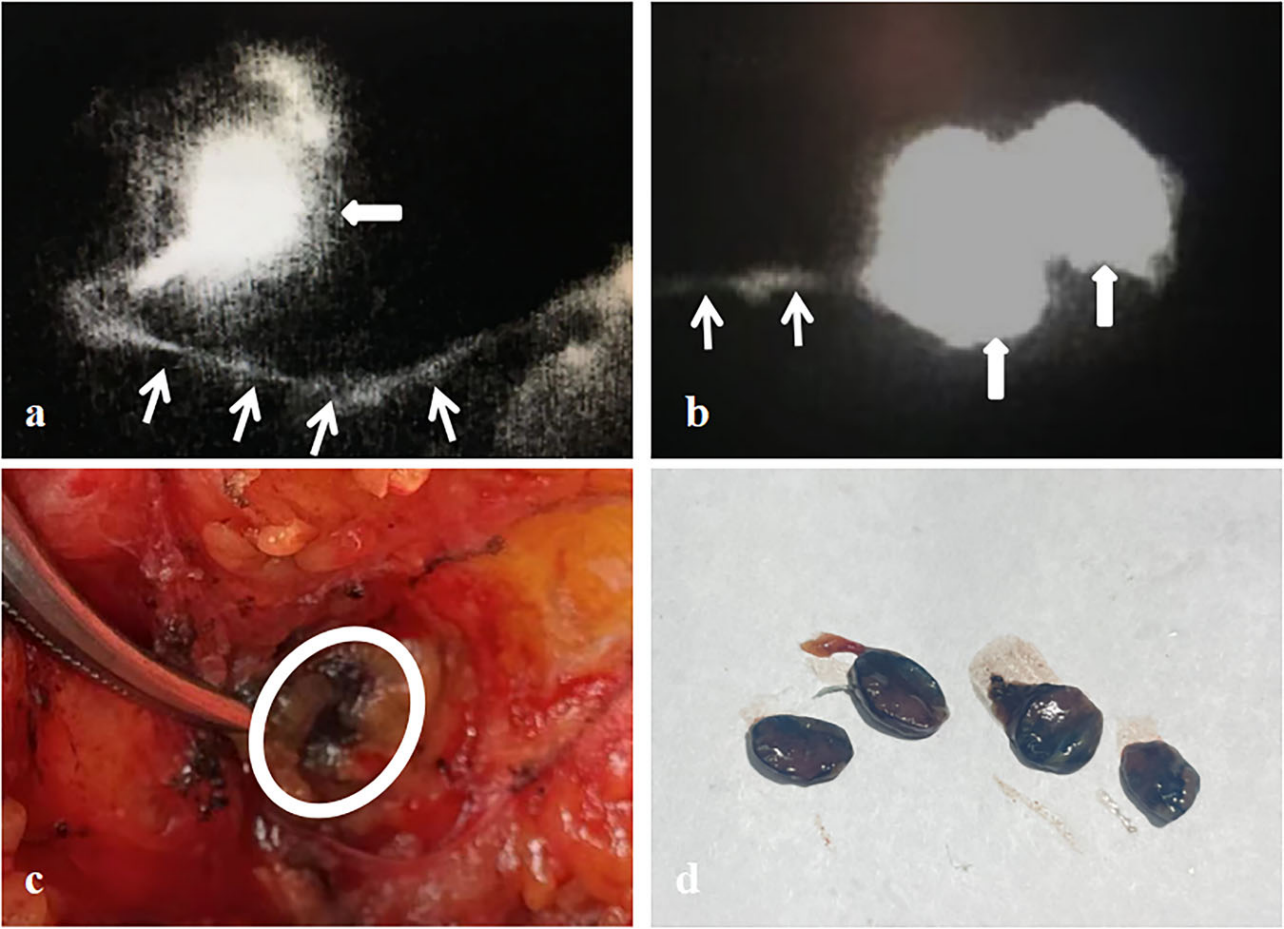
ICG-fluorescent image and intra-operative observations. **a**, **b** The white thin and thick arrows represent the LC and SLN of the ICG-fluorescent image, respectively. **c** The white circle represents the intra-operative blue-stained SLN. **d** Blue-stained SLN profile. ICG, indocyanine green; LC, lymphatic channel; SLN, sentinel lymph node

The CEUS enhancement patterns are depicted in Fig. 4. The CEUS Group successfully identified and localized SLNs in all 76 patients, resulting in a detection rate of 100.0%. The distribution of SLNs detected in the CEUS Group was as follows: 42 patients (55.3%) had one SLN, 20 patients (26.3%) had two SLNs, 12 patients (15.8%) had three SLNs, and 2 patients (2.6%) had four SLNs. The diameter and CEUS pattern of SLN detected by the CEUS Group are detailed in Tables 3 and 4. The two sonographers demonstrated excellent interobserver agreement in CEUS image interpretation, with a kappa value of 0.864 (95% CI: 0.734–0.993). The sensitivity and specificity of the enhancement pattern for diagnosing SLN metastasis were 90.5% (19/21) and 94.5% (52/55), respectively. The ICG-fluorescent image and intra-operative image of the SLN are shown in Fig. 5.

**Table 3:** Short diameter, long diameter versus the pathology results of SLN in CEUS Group

Diameter	Total M (IQR)	SLN negative M (IQR)	SLN positive M (IQR)	z value	*p*-value
Largest SLN					
Short diameter	5.00 (1)	5.00 (1)	6.00 (3)	−2.692	< 0.001
Long diameter	9.00 (6)	8.00 (4)	9.00 (6)	−2.046	0.04
CEUS-suspected SLN					
Short diameter	0.00 (5)	0.00 (0)	6.00 (3)	−7.135	< 0.001
Long diameter	0.00 (8)	0.00 (0)	9.00 (6)	−7.023	< 0.001

Data are shown as median (IQR)

*IQR* interquartile range, *CEUS* contrast-enhanced ultrasound, *SLN* sentinel lymph node, *z* Wilcoxon rank sum test

**Table 4:** CEUS pattern versus the pathology results of SLN

SLN-CEUS pattern	Total (*n* = 76)	SLN negative (*n* = 55)	SLN positive (*n* = 21)	*χ*^*2*^ value	*p*-value
Homogeneous enhancement	54 (71.1)	52 (94.5)	2 (9.5)	53.412	< 0.001
No or heterogeneous enhancement	22 (28.9)	3 (5.5)	19 (90.5)		

Data are shown *n* (%)

*CEUS* contrast-enhanced ultrasound, *SLN* sentinel lymph node, *χ*^*2*^ Pearson χ^2^ test

Correlation analysis revealed a significant association between SLN status and CEUS diagnosis of SLN metastasis (r = 0.838, *p* < 0.001). This study further explored the correlation between SLN metastasis and specific CEUS characteristics. The long and short diameters of the largest SLN, the long and short diameters of the CEUS-suspected SLN, and the SLN enhancement pattern (all *p* < 0.05) were significantly associated with SLN metastasis. Multivariate logistic regression analysis revealed that only the enhancement pattern (odds ratio (OR) = 552.287, 95% CI: 19.059–16,004.187, *p* < 0.001) was independently associated with SLN metastasis, whereas the long and short diameters of the CEUS-suspected SLNs were not statistically significant (*p* > 0.05). The detailed statistical results are presented in Table 5. Collinearity analysis demonstrated variance inflation factors (VIFs) of 3.733 (long diameter) and 4.404 (short diameter) for CEUS-suspected SLNs, with both values below the conventional threshold of 5, demonstrating no significant collinearity.

**Table 5:** The results of the multivariate logistic regression analysis of SLN-CEUS characteristics

Variable	B	SE Coeff	Wald	*p*-value	OR (95% CI)
Short diameter of CEUS-suspected SLN	0.248	0.333	0.554	0.45	1.281 (0.667–2.460)
Long diameter of CEUS-suspected SLN	−0.210	0.175	1.435	0.23	0.811 (0.575–1.143)
SLN-CEUS pattern	6.314	1.718	13.513	< 0.001	552.287 (19.059–16,004.187)

*B* regression coefficients, *SE Coeff* standard error of coefficient, *OR* odds ratio, *CI* confidence interval, *SLN* sentinel lymph node, *CEUS* contrast-enhanced ultrasound

**Fig. 6:**
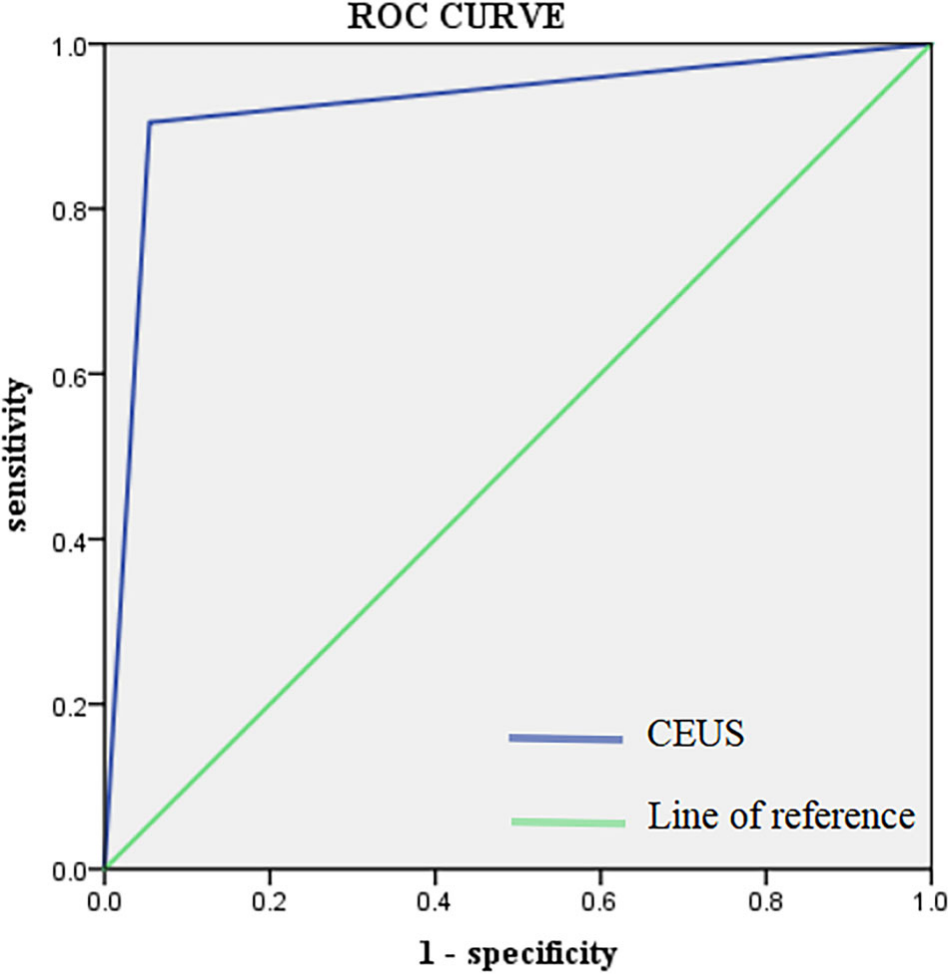
Receiver-operating characteristic curve (ROC) for CEUS in predicting metastasis in SLN, the AUC = 0.925. CEUS, contrast-enhanced ultrasound; SLN, sentinel lymph node; AUC, area under the ROC

The sensitivity, specificity, positive predictive value, and negative predictive value of CEUS for diagnose SLN metastasis were 0.905 (95% CI: 0.6817–0.9833), 0.945 (95% CI: 0.8393–0.9858), 0.863 (95% CI: 0.6403–0.9641), and 0.963 (95% CI: 0.8616–0.9935), respectively, with a FNR of 9.5%. The AUC for CEUS was 0.925 (Fig. 6).

## Discussion

Accurate preoperative identification and localization of SLNs have become increasingly critical in clinical practice. CEUS has emerged as a promising non-invasive modality for preoperative SLN detection and assessment. In this study, we propose an integrated SLN mapping strategy that combines preoperative CEUS, lymph node MB injection, and intra-operative ICG injection. This multimodal approach achieved a 100% SLN detection rate. Notably, the CEUS Group demonstrated a significant reduction in the number of SLNs excised compared to the Blue Staining Group (z = 8.499, *p* < 0.001), thereby minimizing unnecessary non-SLN resection. Furthermore, CEUS enhancement patterns (odds ratio (OR) = 552.287, 95% CI: 19.059–16,004.187, *p* < 0.001) exhibited a strong independent association with SLN metastasis. These findings underscore the diagnostic efficacy of CEUS in SLN metastasis evaluation, supported by its strong predictive performance (AUC = 0.925). Collectively, our results highlight the clinical utility of CEUS in refining SLN mapping protocols and reducing overtreatment through precise preoperative planning.

CEUS process involves injecting UCA intracutaneously through the areola, which then travels along LCs to the SLNs, facilitating their accurate identification and mapping. In this study, 76 patients received UCA injections at the 12 o’clock position of the areola, achieving a 100.0% SLN detection rate, comparable to the rates reported by Li et al [14] and Zhong et al [20]. The areola lymphatic plexus, a dense and interconnected network under the nipple areola, allows UCAs injected at any point around the areola to reach the SLNs. Furthermore, single-point UCA injection for SLN-CEUS significantly reduces patient pain without compromising the detection rate.

Our results align with those of previous studies [4, 21–23], showing that the average number of SLNs identified per patient in the CEUS Group was 1.66, with a median of 1. This is notably fewer compared to the Blue Staining Group, which identified an average of 4.22 SLNs per patient, with a median of 4. The main reason for this difference may be that CEUS enables real-time visualization and precise localization of the enhanced true-SLNs. In contrast, the time required to identify blue-stained SLNs following intra-operative intracutaneous injection of MB exhibited variability. Prolonged intervals may lead to dye migration to non-SLNs, increasing the risk of misidentification and unnecessary removal, which can result in more excised lymph nodes and higher postoperative complications [24–26].

CEUS has become a vital tool for preoperative prediction of SLN metastasis, providing important prognostic insights that inform surgical decision-making. Homogeneous enhancement indicates no SLN metastasis, while heterogeneous or no enhancement suggests the presence of metastasis [15, 19]. In this study, the sensitivity and specificity of CEUS lymph node enhancement pattern in diagnosing SLN metastasis were 90.5% and 94.5%, respectively, consistent with a recent meta-analysis [27]. Multivariate logistic regression analysis revealed that only the enhancement pattern was independently related to SLN metastasis [3]. The AUC for CEUS in diagnosing SLN metastasis was 0.925. These results indicate that CEUS demonstrates high diagnostic efficacy for detecting SLN metastasis by integrating enhancement patterns with the long and short diameter characteristics of the SLNs.

In two cases, hyperplasia and lymphatic sinus dilation in SLNs appeared as heterogeneous enhancement on CEUS, leading to misinterpretation as metastasis. These conditions can be differentiated from true metastasis by their distinct lymphatic portal architecture, uniform thickened cortical echo, and the absence of calcification and necrosis. Additionally, one patient who had undergone NAC was incorrectly diagnosed with metastasis. This misdiagnosis may have resulted from the chemotherapeutically induced slenderization of LC, which obstructed the passage of the UCA, resulting in the absence of enhancement on CEUS imaging [28]. In two patients with confirmed SLN metastasis, CEUS demonstrated consistent enhancement, but these isolated tumor cell clusters were less than 2 mm in diameter. This small size can affect diagnostic accuracy, as such micrometastases may not be easily detectable, consistent with literature reports [8, 29]. This limitation could lead to false negatives and influence clinical decisions regarding adjuvant therapy for breast cancer [30]. Future research should aim to enhance the diagnostic accuracy of micrometastases through the integration of molecular imaging techniques.

The FNR is a critical metric for evaluating the accuracy of SLNB. An FNR of less than 10.0% is deemed acceptable for SLNB [31], and our study’s FNR was 9.5% (2/21), meeting this criterion. SLN-CEUS can accurately locate SLNs and meet the FNR requirement while avoiding the excision of non-SLNs, offering a promising approach in clinical application.

This study established a direct correlation between CEUS results and SLNB surgical procedures. After identifying SLNs using CEUS, a US-guided intra-lymph node MB injection was performed, allowing for direct observation of blue-stained SLNs during surgery. This approach not only confirmed the accuracy of CEUS in identifying SLNs but also enabled clinicians to quickly and precisely localize them, thereby reducing operation time and minimizing trauma associated with the excision of non-SLNs.

## Limitations

The study’s sample size was limited, which may have implications for the interpretation of the results and warrants further investigation in future studies. Besides, as this was a single-center study, our findings require validation through larger, multi-center investigations to ensure generalizability. Additionally, the clinical evidence is limited by insufficient external validation and a lack of comparison with gold-standard radioisotope tracing. Furthermore, the operator-dependent nature of CEUS necessitates comprehensive training programs to ensure consistency and facilitate widespread clinical implementation. We also recognize that unaccounted variables, including body mass index, ultrasound contrast agent uptake, and previous surgeries, may affect CEUS accuracy, as noted in our limitations.

## Conclusions

CEUS enhancement pattern is helpful to determine the SLN metastasis. Preoperative CEUS, combined with MB injections into lymph nodes and intracutaneous ICG injections during surgery, provides a reliable method for accurately localizing SLNs and minimizes the risk of excising non-SLNs.

## Abbreviations

ALN: Axillary lymph node
ALND: Axillary lymph node dissection
AUC: Area under the curve
BD: Blue dye
CEUS: Contrast-enhanced ultrasound
FNR: False negative rate
ICG: Indocyanine green
LC: Lymphatic channel
MB: Methylene blue
NAC: Neoadjuvant chemotherapy
RI: Radioisotope
ROC: Receiver operating characteristic curve
SLN: Sentinel lymph node
SLNB: Sentinel lymph node biopsy
UCA: Ultrasound contrast agent
US: Conventional ultrasound
